# Systemic Drug Effects in Vortioxetine-Induced Time-Series Datasets

**DOI:** 10.3390/ijms27136058

**Published:** 2026-07-06

**Authors:** Shinuk Kim

**Affiliations:** College of Gyedang General Education, Sangmyung University, Cheonan 31066, Chung-Nam, Republic of Korea; kshinuk@smu.ac.kr

**Keywords:** drug effect, systemic model, time-series data, gene–gene interaction, ERBB signaling pathway, GLIOMA pathway

## Abstract

This paper introduces an approach for inferring the gene regulatory networks in vortioxetine-induced glioblastoma cells to investigate vortioxetine’s systemic effects. The approach uses an ordinary differential equation (ODE)-based inverse problem to evaluate the drug-induced gene interactions within the GLIOMA and ERBB pathways, which are deeply intertwined in cancers, by using time-series datasets. Time-series datasets were generated in triplicate at 0, 3, 6, 9, 12, and 24 h. The results of the ERBB pathway confirmed that *PIK3R5* was commonly activated, while *JUN*, as a proto-oncogene in glioblastoma, was inhibited by genes across all three datasets. In particular, *PIK3R5* was commonly activated by *PAK6* in all three datasets. The results of the GLIOMA pathway confirmed that *CALML6* was commonly activated, while *CDK4* and *CCND1*, which are mostly overexpressed in human cancers, were inhibited across all three datasets. Additionally, an analysis of the independent datasets generated at 6 and 22 h after the vortioxetine injection identified the most distinct variable genes between the two time points: *CRK* (1.96) and *JUN* (−3.02) for the ERBB signaling pathway, and *BRAF* (1.30) and *MAP2K2* (−1.92) for the GLIOMA pathway. We conclude that vortioxetine, an antidepressant, decreases *JUN*, a proto-oncogene involved in the ERBB signaling pathway, and *CCND1*, another proto-oncogene involved in the GLIOMA pathway, over time in glioblastoma cells.

## 1. Introduction

Vortioxetine is a multimodal antidepressant that targets both serotonin receptors and transporters and is primarily used to treat major depressive disorder though a dual pharmacological strategy. Despite its clinical use, the exact mechanism of action remains unclear. Recent research utilizing WST-8 cell counting assays and clonogenic assays has demonstrated that vortioxetine treatment inhibits both the short-term viability and long-term survival of glioblastoma cells, as well as their migration. These findings suggest that vortioxetine may be a promising candidate for drug repurposing [1].

Glioblastoma (GBM) is the most aggressive heterogeneous brain cancer, with median survival of 15~16 months and a 5-year survival rate of approximately 5%~10% [2]. GBM is classified as either primary, affecting elderly patients de novo, or secondary, mainly affecting younger patients [3]. The standard therapies for this condition have limited success due to the blood–brain barrier restricting tumor accessibility, the presence of treatment-resistant GBM stem cells, and the absence of clinically predictive patient model systems [4]. Understanding the systemic effects of relevant drugs is crucial for patient safety, effective dosing, and predicting adverse reactions [5].

Another obstacle to systemic therapy is the absence of molecular datasets over time. Approaches that involve gene expression datasets over time provide a clear picture of functional gene interactions in the corresponding networks. In this context, inverse algorithms using time-series datasets have been adopted to infer systemic gene regulatory networks [6,7,8]. Yet, most biological laboratory experiments produce static datasets or limited time points, making it challenging to understand systemic molecular changes. This study used an inverse algorithm for a gene regulatory network to investigate the systemic effects of a drug over time. Recently, Lee et al. [5] generated time-series datasets of human cells. In that study, they analyzed three datasets containing 67 neuroactive anticancer drugs and found that the most active drug was vortioxetine, an antidepressant. Based on these results, Lee et al. [5] obtained RNA-seq time-series datasets in triplicate at 0, 3, 6, 9, 12, and 24 h from the glioblastoma cell line LN229 treated with vortioxetine. This study utilized these datasets submitted to NCBI under the designation GSE214968 (https://www.ncbi.nlm.nih.gov/geo, accessed on 16 January 2025). Lee et al. [5] also deposited datasets under the designation GSE214965, which comprised 20 drugs at two time points (6 and 22 h) with two GBM cell lines (LN229), and the experiment was replicated twice. Despite advanced measurement technology, laboratory noise and time delays persisted. Consequently, the datasets were partitioned across experiments to ensure reproducible findings.

In this study, we restricted the gene sets of the GLIOMA pathway and the ERBB signaling pathway to investigate the effects of the drug treatment on gene interactions. The GLIOMA pathway is a gene set associated with the development of glioblastoma (GBM) driven by disruptions in multiple signaling pathways, including PI3K/AKT/mTOR, Wnt, NF-κB, and TGF-β [3,9]. The ERBB signaling pathway is known as part of the receptor tyrosine kinase (RTK) signaling pathway, which plays a crucial role in the development and progression of GBM [10,11].

For the computational analysis of the mathematical model, we employed ordinary differential equations (see Methods for more details) [6,7], which were fitted to identify interactions using time-series datasets. Based on this framework, we formulated the inference problem using a mathematical optimization method called the Broyden–Fletcher–Goldfarb–Shanno (BFGS) method [12]. We retrieved the top 10 positive and top 10 negative interactions utilizing the time-series datasets by applying a computational model known as an inverse method to three distinct datasets from triplicate trials. Using the criterion of an absolute value of 1, we contrasted basic statistical analyses on two-time-point datasets to identify genes that were either over- or underexpressed.

## 2. Results

We tested the program using the *L*2 objective function in the inverse algorithm based on the two pathways involved in GBM cancer: the ERBB signaling pathway, which is involved in cancer development, and the GLIOMA pathway, which is deeply intertwined in the AKT/PI3K/mTOR pathways. The results of drug-effect gene interactions for each dataset are shown below.

### 2.1. The ERBB Pathway for GSE214968

**Figure 1:**
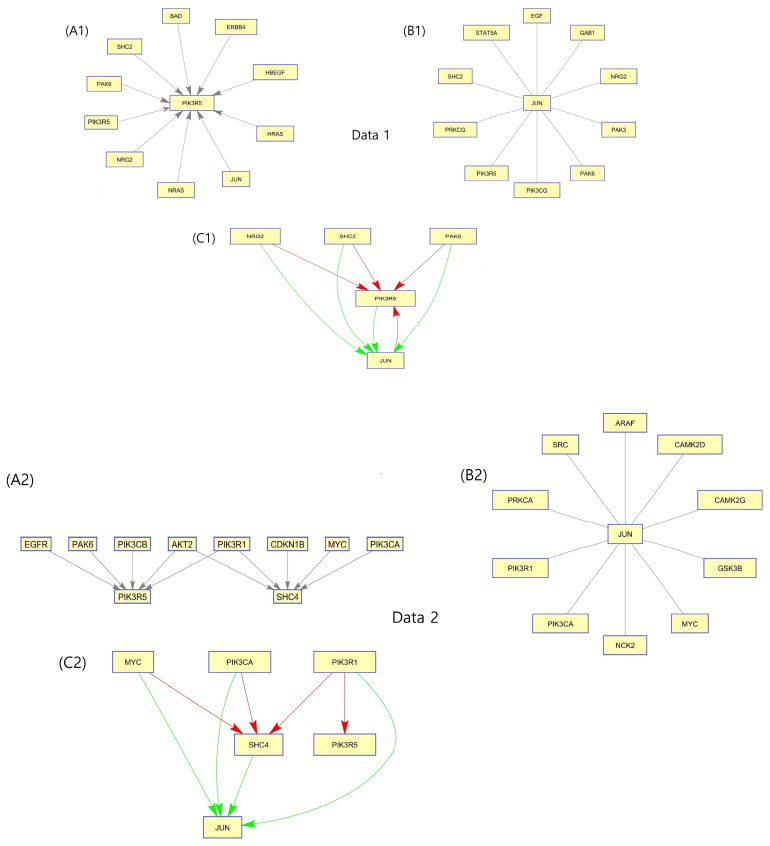

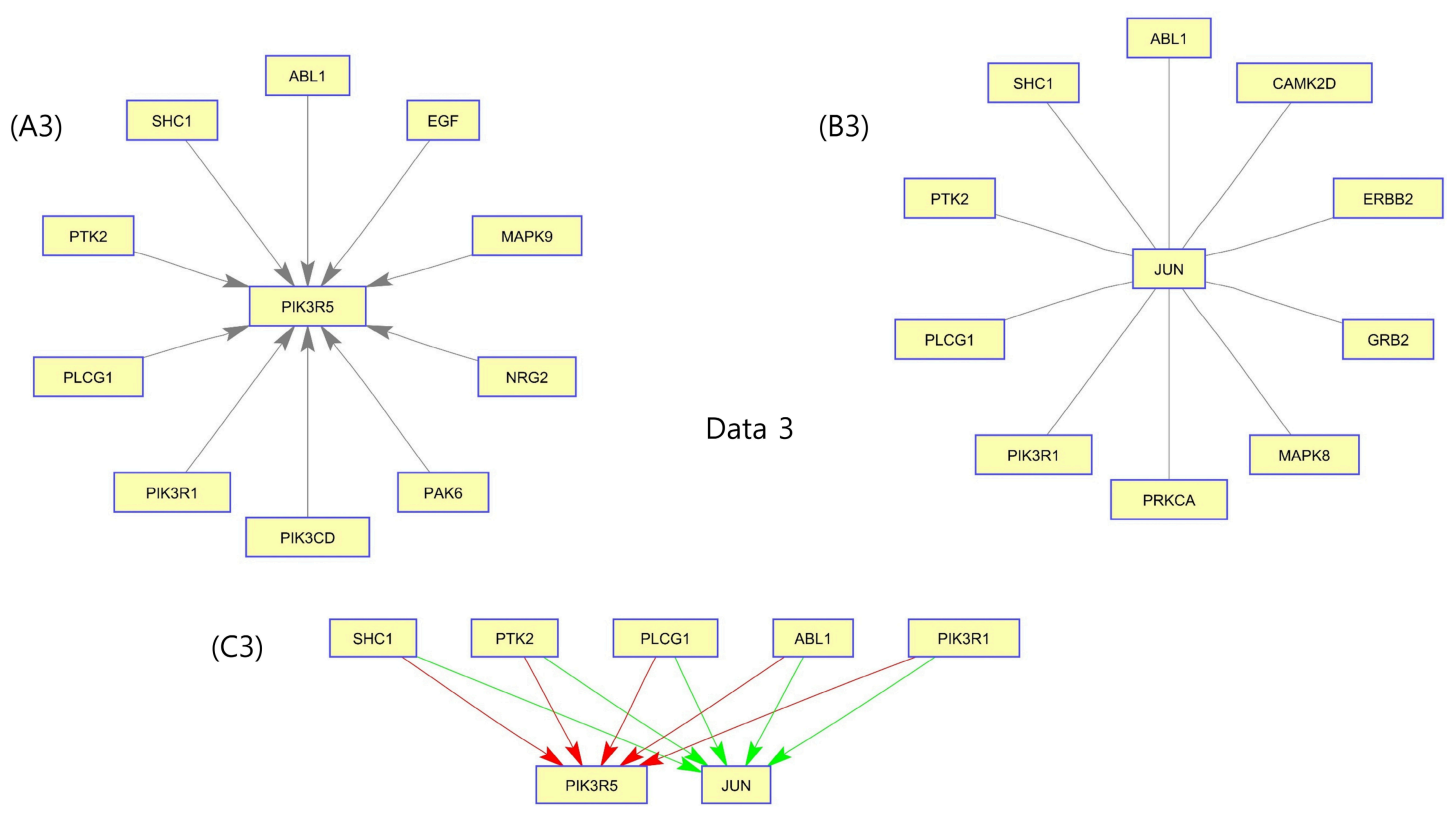
The results for data1 (**A1**,**B1**,**C1**; upper panel), data2 (**A2**,**B2**,**C2**; middle panel), and data3 (**A3**,**B3**,**C3**; lower panel). (**A1**,**A2**,**A3**) represent the top 10 positive gene interaction in data1, data2 and data3 respectively, and the arrows indicate the direction. (**B1**,**B2**,**B3**) represent the top 10 negative genes interactions in data1, data2 and data3, respectively. (**C1**,**C2**,**C3**) represent the combination of activated and inhibited interactions of the ERBB pathway in data1, data2 and data3, respectively, where red arrows signify activation of a molecule, and green arrows indicate inhibition.

Concerning the triplicate experiments, the datasets were separated by plate and named data1, data2, and data3, respectively. After execution, the top 10 positive and the top 10 negative interactions were identified for each dataset. The results are shown in Figure 1 (data1, data2 and data3).

As illustrated in Figure 1 (A1–A3), the results for data1 and data3 show that the top 10 genes activate *PIK3R5* (including *SHC4* for data2), a component of PI3K, which is often linked to therapeutic resistance and cell survival [9]. *PAK6*, which activates the p21 tumor suppressor [13,14], activates *PIK3R5* in all three datasets. *NRG2*, a solid tumor malignancy oncogene [15], activates *PIK3R5* in data1 and data3. *PIK3R1*, a member of the PI3K family that is associated with cancer development and serves as a therapeutic target of the dysregulated PI3K pathway in cancer treatment, activates *PIK3R5* in data2 and data3. Despite the use of the PI3K family in cancer treatment, the PI3K signaling pathway has not been fully elucidated due to its extreme complexity, its high redundancy, and the development of rapid compensatory feedback loops that allow cancer cells to bypass a blockade [16,17]. In this regard, we uncovered an interesting finding that *PIK3R1* activates *PIK3R5*. This is clinically significant because *PIK3R5*, a regulatory subunit of PI3K, suppresses the AKT/mTOR pathway, and its downregulation leads to pathway activation, promoting cancer growth and survival [18].

Conversely, as shown in Figure 1 (B1–B3), *JUN*, a proto-oncogene that is part of the signaling network that promotes proliferation and survival in cancer cells with active ERBB signaling [19], is inhibited by the other top 10 genes in all datasets (data1, 2, and 3). Suppressing *JUN* expression is particularly noteworthy given that *JUN*, a proto-oncogene, is linked to breast cancer and glioma susceptibility [19,20] in GeneCards and MalaCards [21,22] as of July 2025. In addition, *PRKCA*, *CAMK2D*, and *PIK3R1* commonly inhibit *JUN* in both data2 and data3. However, data1 and data2 do not share a negative gene that inhibits *JUN*.

**Figure 2:**
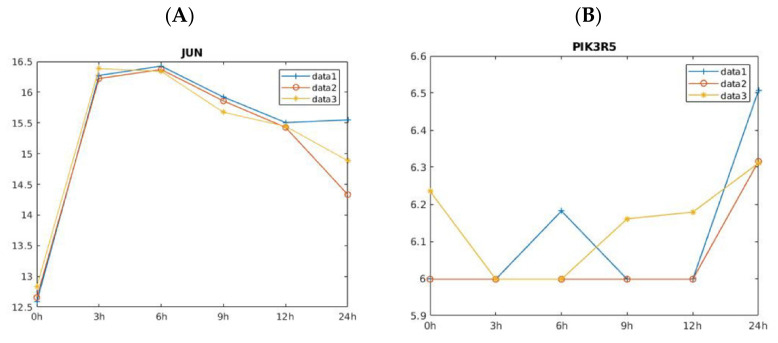
(**A**) Expression levels of JUN and (**B**) expression levels of PIK3R5 at 6 time points.

Figure 2 shows the expression levels of the repressor gene *JUN* and the activator gene *PIK3R5* from the three datasets. It indicates that the drug’s effect on *JUN* consistently increases at 3 h and then decreases over time across all three datasets. This suggests that the drug is likely to affect not only *JUN* but also genes that inhibit it systemically.

Interpreting changes in the expression level of *PIK3R5* over time is complicated. The expression level of *PIK3R5* begins to increase after 6 h in data 3 and after 12 h in data1 and data2, and it remains overexpressed in all datasets after 24 h.

### 2.2. The GLIOMA Pathway for GSE214968

#### 2.2.1. Results for Data1

**Figure 3:**
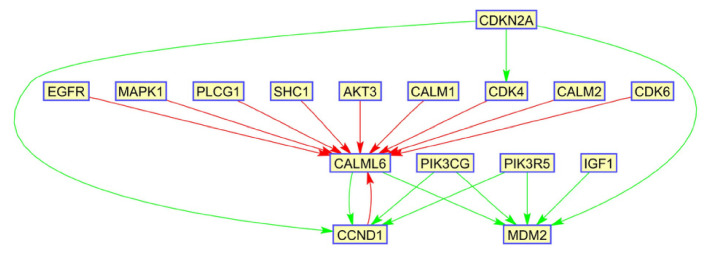
The combination of activated (red arrows) and inhibited (green arrows) interactions for data1.

Figure 3 shows the top 10 activated interactions (red arrows) and the top 10 inhibited interactions (green arrows). *CALML6*, a gene that is associated with cancer and is either over- or underexpressed in cancer development [23], is activated by *PLCG1*, *SHC1*, *AKT3*, *CALM1*, *CALM2*, *CDK4*, *CDK6*, *EGFR*, *MAPK1* and *CCND1*. *MDM2*, a pro-oncogene destroying tumor suppressor p53 [23,24], and *CCND1*, an oncogene expressed in human breast cancer [25], are inhibited by *PIK3CG*, *PIK3R5*, *CALML6* and *CDKN2A*. *CDK4* is inhibited by *CDKN2A* [26], a tumor suppressor gene.

#### 2.2.2. Results for Data2

**Figure 4:**
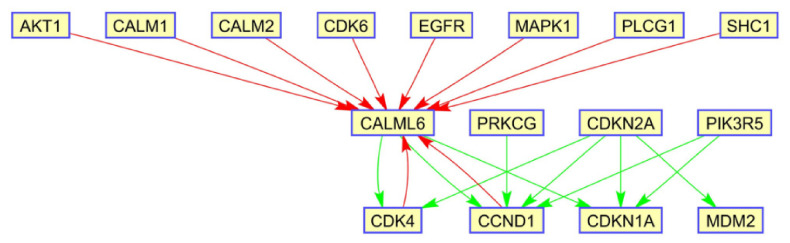
The combination of activated (red arrows) and inhibited (green arrows) interactions for data 2.

Figure 4 shows the top 10 activated (red arrows) and the top 10 inhibited (green arrows) interactions. *CALML6*, a biomarker of several cancers, especially an inflammation-associated gene in papillary thyroid cancer [23], is activated by *AKT1*, *CALM1/2*, *CDK4/6*, *EGFR*, *MAPK1*, *PLCG1*, *SHC1*, and *CCND1*. *CDKN2A* inhibits *CDK3*, *CCND1*, *CDKN1A*, and *MDM2*. *PIK3R5* inhibits *CCND1* and *CDKN1A*. *PRKCG* inhibits *CCND1*. *CALML6* inhibits *CDK4*, *CCND1*, and *CDKN1A*.

#### 2.2.3. Results for Data3

**Figure 5:**
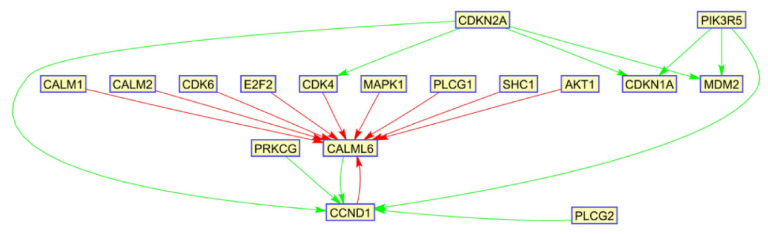
The combination of activated (red arrows) and inhibited (green arrows) interactions for data 3.

Figure 5 illustrates the top 10 activated (red arrows) and the top 10 inhibited (green arrows) interactions. *CALML6* is overexpressed in 11 tumors, including liver carcinoma, bladder carcinoma, lung adenocarcinoma, and prostate, colon, breast, kidney, stomach, uterine, and rectal carcinoma, and underexpressed in three tumors: thyroid cancer, pancreatic adenocarcinoma, and renal cell carcinoma [23]. In the GBM cell line used in this study, we confirmed that *CALML6*, which is involved in several aspects of tumorigenesis, is activated by the proteins *PLCG1*, *CDK4*, *CDK6*, *SHC1*, *AKT1*, *CALM1*, *CALM2*, *E2F2*, *MAPK1*, and *CCND1*. The inhibited genes are *CDK4*, *CCND1*, *MDM2*, and *CDKN1A*. The interactions are similar to but not the same as those in the results for Data 2. Instead of *PRKCG* in data2, both *PLCG2* and *PRKCG* inhibit *CCND1* in data3.

The results of gene interactions based on the GLIOMA pathway are highly consistent. This is because *CALML6*, a potential biomarker for thyroid cancer [23,27], is activated by eight genes that are present in all three datasets: *CALM1*, *CALM2*, *SHC1*, *PLCG1*, *MAPK1*, *CDK4*, *CDK6*, and *CCND1*. Furthermore, *CCND1*, which is overexpressed in human cancer cells [25,28], is inhibited by *CALML6*, *PIK3R5*, and *CDK4* in data3, as well as by *CDKN2A* in all three datasets.

### 2.3. Results for GSE214965 in Two-Time-Point Datasets

#### 2.3.1. Based on the ERBB Signaling Pathway

Eight vortioxetine-induced samples were extracted from 20 distinct drugs at two time points in the downloaded datasets, utilizing 71 ERBB pathway genes from GSE214965. Using the two time points, 6 and 22 h, we calculated positive (increased) and negative (decreased) changes over time in two different cell lines, as shown in Table 1. As a result, we obtained nine overexpressed genes and 11 underexpressed genes based on an arbitrary criterion of a 2-fold change or greater.

The largest change in the positive genes occurred in *CRK* (1.96), an oncogene [29], and the largest change in the negative genes occurred in *JUN*, a proto-oncogene [19]. Among the overexpressed genes, *NRG2* is described in two cases: as a tumor suppressor underexpressed in breast cancer patients [30,31] and as an oncogene overexpressed in GBM [32]. However, this study found that *NRG2* was overexpressed. Thus, it appears that the role of *NRG2* is context- and type-specific, as its function shifts between acting as a tumor promoter and a tumor suppressor depending on the tissue and type of cancer.

Here, we found that *CBLB*, a tumor suppressor gene [33], was overexpressed, suggesting that the drug affects GBM through this gene. The *AKT2* [34], *ERBB3* [35], and *BRAF* [36] genes, which are proto-oncogenes, are found in the overexpressed column, suggesting that the drug is not given sufficient time to affect these genes for GBM treatment. However, the drug affects the tumor suppressor gene *CRK* for the treatment of GBM. Among the underexpressed genes, *CAMK2D* is associated with a neurodevelopmental disorder [37] and is known as a tumor suppressant gene. *NRG1*, which is known as an oncogenic and target gene for cancer therapy, appears in the underexpressed column, while *NRG2*, which operates as a tumor suppressor, appears in the overexpressed column [38].

#### 2.3.2. Based on the GLIOMA Pathway

The GLIOMA pathway in the GSE214965 datasets contains 50 out of the total 64 genes. Table 2 presents the results of expression levels calculated using Equation (3). We obtained five overexpressed genes and eight underexpressed genes based on a criterion of an absolute value difference of ≥1. The most underexpressed gene was *MAP2K2* (−1.92), and the most overexpressed gene was *BRAF* (1.03), both of which are involved in various cancer types [36,39]. In the results, *JUN*, a highly expressed gene in the ERBB pathway, was not observed here because 21 of the 64 GLIOMA pathway genes were not in the ERBB pathway.

## 3. Discussion

Based on the gene sets associated with the ERBB signaling pathway and the GLIOMA pathway, we identified the top 10 positive and negative interactions from time-series datasets of vortioxetine-induced gene interactions. The ERBB signaling pathway analysis of all three vortioxetine-induced datasets showed that *PIK3R5*, an oncogene, is activated by 10 genes, whereas *JUN*, a proto-oncogene, is inhibited by other genes. However, the activating genes and inhibiting genes are inconsistent across all three datasets, except for *PAK6*. *PAK6* is a gene that plays a complex role in activating the tumor suppressor gene p2, which suppresses prostate tumor growth [40] and is overexpressed in several cancers [41]. This study found that *PAK6* activates *PIK3R5* in all three datasets. *NRG2* activates *PIK3R5* in data1 and data3. *PIK3R1* activates *PIK3R5* in data2 and data3. However, there are no common positive interactions between data1 and data2.

*CAMK2D*, *PIK3R1*, and *PRKCA* inhibit *JUN* in data2 and data3, but there are no overlapping negative interactions between the other datasets. The SHC family of oncogenes [42] was identified as either activating *PIK3R5* or inhibiting *JUN* in all datasets. Taken together, these results indicate that the antidepressant drug vortioxetine inhibits the oncogene *JUN* and activates the oncogene *PIK3R5* in the ERBB signaling pathway.

The GLIOMA pathway analysis of all three vortioxetine-induced datasets showed that *CALML6* is commonly activated by eight genes, *PLCG1*, *CDK4*, *CDK6*, *CCND1*, *SHC1*, *CALM1*, *CALM2*, and *MAPK1*, the latter being an oncogene associated with cell proliferation and malignancy [43]. *CCND1* was commonly inhibited by *CALML6*, *CDKN2A*, and *PIK3R5* in all three datasets. *CALML6,* a biomarker for several cancers, is overexpressed in 11 tumors—liver carcinoma, bladder carcinoma, lung adenocarcinoma, lung squamous cell carcinoma, prostate adenocarcinoma, colon adenocarcinoma, breast invasive carcinoma, kidney renal clear-cell carcinoma, stomach adenocarcinoma, uterine carcinoma, and rectal adenocarcinoma—and underexpressed in three tumors—thyroid cancer, pancreatic adenocarcinoma, and renal cell carcinoma [23].

In all three datasets, *CALML6* and *CCND1* regulate each other: *CALML6* activates *CCND1*, while *CCND1* inhibits *CALML6*. Furthermore, a regulatory network exists in which *CDKN2A* inhibits *CDK4*, *CDK4* activates *CALML6*, and *CALML6* both activates and is inhibited by *CCND1*. *CAMK2D*, a gene involved in brain and heart development and acting as a scaffold for tumor-suppressing complexes, is underexpressed in both the ERBB signaling pathway and the GLIOMA pathway in the two-time-point datasets.

## 4. Materials and Methods

### 4.1. Materials

We tested two RNA-seq datasets (GSE214968 and GSE214965) for GBM downloaded from https://www.ncbi.nlm.nih.gov/geo/query/acc.cgi?acc=GSE214968 (accessed on 16 January 2025), https://www.ncbi.nlm.nih.gov/geo/query/acc.cgi?acc=GSE214965 (accessed on 16 January 2025). The GSE214968 datasets were generated by Lee [5], designed for LN229 GBM cells at baseline 0 and at 3, 6, 9, 12, and 24 h time points after vortioxetine treatment, with 16,674 genes and three replicates, leaving 18 samples. The second dataset, GSE214965, was also designed for the LN299 cell line and included 160 samples from two different cell lines at two time points (6 and 22 h), 20 drugs, 12,738 genes, and two replicate experiments. From the two datasets, only the vortioxetine drug-induced datasets were extracted.

The ERBB signaling pathway, a part of the RTK signaling pathway, plays an important role in the development and progression of various cancers, especially GBM [10,11,44]. The ERBB signaling pathway contained 87 genes downloaded from the KEGG pathway [45]. After filtering, the GSE 214968 dataset contained 83 ERBB pathway genes, and the GSE214965 dataset contained 71 ERBB pathway genes. The GLIOMA pathway associated with the development of GBM, which is complicated and intertwined with the PI3K/AKT/mTOR, Wnt, NF-κB, and TGF-β signaling pathways [3], contained 61 genes out of 64 downloaded from the KEGG pathway.

### 4.2. Methods

We used two different methods by data type: an inverse algorithm for time-dependent datasets and a statistical method for datasets with two time points using Matlab_R2021b (Mathworks, Inc. Natick, MA USA).

#### 4.2.1. Inverse Algorithm Method for GSE214968 Time-Dependent Datasets

**Figure 6:**
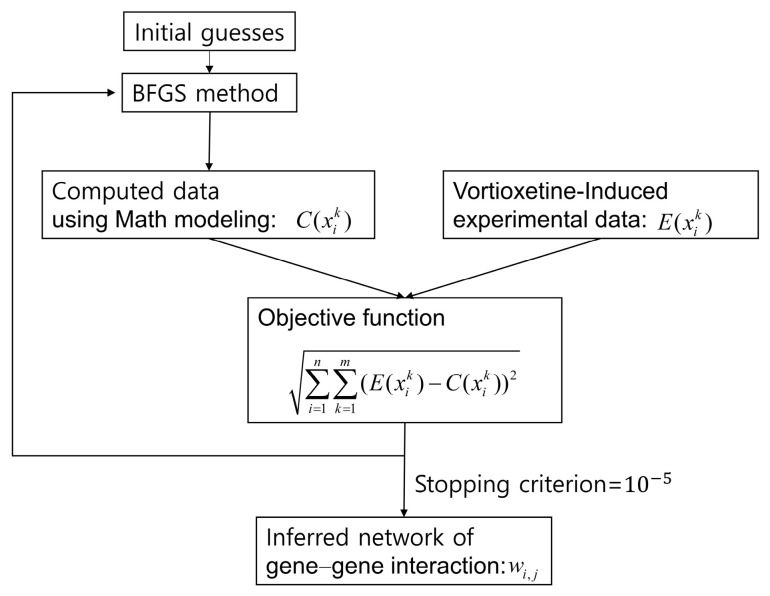
A flowchart of the computational method.

This method comprises three parts: a direct solver, an optimization routine, and an objective function [6]. A direct solver is used to generate computational datasets containing unknown parameters, such as gene interactions. An optimization method was used to make the error norm approach zero by determining the unknown parameters of the computational data. The BFGS scheme was used; this involves estimating the inverse quasi-Newton matrix to determine the next iteration step [12]. A flowchart of the three-part method is shown in Figure 6 below.

The direct solver generates computational datasets using finite-difference approximations, as shown below in Equation (1). $x_{i}^{k}$ represents data for gene *i* and timeline *k*, and *w_i,j_* represents the interaction between gene *i* and gene *j*. If *w_i,j_* is positive, then gene *j* activates gene *i*; if *w_i,j_* is negative, then gene *j* inhibits gene *i*. *dt* refers to the partial derivative with respect to time [6].

(1) $$x_{i}^{k+1}=x_{i}^{k-1}+dt\sum_{j=1}^{m}w_{i,j}x_{j}^{k-1}$$

The objective function was used to determine the global minimum of the error norms by integrating the differences between the computational data, including the interaction parameters, and the experimental data. Hence, the objective function provided a stopping criterion for the iterations. In this study, both L1 and L2 norms were evaluated. The L1 norm exhibited unstable performance and failed to converge, whereas the L2 norm demonstrated superior performance. Therefore, we employed the L2 norm as shown below in Equation (2):

(2) $$L_{2}=\sqrt{\sum_{i=1}^{n}\sum_{k=1}^{m}{\left(E_{i,k}-C_{i,k}\right)}^{2}},$$

where $E_{i,k}$ represents the experimental time-dependent datasets for the *i*th gene and *k*th time point, and $C_{i,k}$ represents the numerical datasets including the interaction parameters. For program implementation, unbiased zero values were selected as initial guesses, and the stopping criterion was defined as.

#### 4.2.2. Sample Comparison for GSE214965 Two-Time-Point Datasets

In this study, second datasets were generated at 6 and 22 h after induction with vortioxetine. Repeated measurements were performed in the laboratory using two different LN299 cell lines, yielding four data points at each time point. Therefore, the data for each time point were averaged and analyzed to determine the differences between the 22 h and 6 h time point datasets. *DEG_i_*, as shown below in Equation (3), is the difference between the two time points for each gene:

(3) $$DEG_{i}=G_{i}^{22h}-G_{i}^{6h},$$

$$G_{i}^{22h}=mean\sum_{k=1}^{4}x_{i,k}^{22h},\;G_{i}^{6h}=mean\sum_{k=1}^{4}x_{i,k}^{6h}$$

where $G_{i}^{22h}$ is the mean expression of the ith gene at 22 h, $G_{i}^{6h}$ is the mean expression of the ith gene expression at 6 h, and *DEG_i_* is the difference over time for gene i. If k is 1 or 2, then $x_{i,k}^{j}$ is data generated from the two cell lines; if *k* is 3 or 4, it is a replicated dataset.

## 5. Conclusions

Despite the remarkable advancements in large-scale gene expression technologies, experimental noise, time delays, and cohort noise persist. An ODE-based mathematical method may contribute to improving cancer drug research by facilitating high-throughput multi-omics data analysis to predict drug resistance, find new therapeutic targets, and ease drug repurposing. In this study, we proposed drug-induced gene interactions within the ERBB signaling pathway and the GLIOMA pathway, utilizing two distinct methodologies with two separate time-dependent datasets.

First, we employed an inverse algorithm to investigate systemic gene interactions indicating drug efficacy using time-series datasets generated at 0, 3, 6, 9, 12, and 24 h in a vortioxetine-treated GBM cell line. Second, we employed a statistical method on replicated datasets at 6 and 22 h.

For the ERBB signaling pathway, we concluded that the proto-oncogene *JUN* was suppressed, while the oncogene *PIK3R5* was expressed in all replicated datasets. According to earlier research, both *JUN* and *PIK3R5* are cancer-related genes and are especially overexpressed in cancerous tissues. In this regard, our study findings indicate that the proto-oncogene *JUN* is inhibited by other genes, suggesting that the drug influences GBM via *JUN*. The oncogene *PIK3R5* is activated by other genes, suggesting that the drug is not effective for treating GBM. While prior research [46] indicates that vortioxetine suppresses that PI3K pathway, we found that this drug does not inhibit *PIK3R*, which is frequently active in GBM. Consequently, it is plausible that the inhibition of the PI3K pathway caused by this medication might be due to a different gene, rather than *PIK3R*.

The GLIOMA pathway analysis indicated that *CALML6* is commonly activated by other genes across all three datasets, whereas *CCND1* and *MDM2* are commonly inhibited by other genes in the same datasets. These gene interactions were not observed in the ERBB signaling pathway. In addition, eight genes across all three datasets were found to commonly activate *CALML6*, a gene previously shown to be overexpressed or underexpressed in various tumors. This clearly demonstrates that vortioxetine consistently affects *CALML6*, suggesting that the drug treats GBM by activating *CALML6* over time via the GLIOMA pathway.

Both *CCND1*, an oncogene that is inhibited by the tumor suppressor miR-206 [47], and *MDM2*, which acts as an oncogene by binding to the tumor suppressor protein p53 [48], are inhibited by other genes across all three datasets. This implies that the drug consistently treats GBM by inhibiting these oncogenes through the GLIOMA pathway over time. *CDK4/6* are known to be key enzymes in cancer cell division, and inhibiting these kinases is used to treat a variety of cancers [49]. *CDKN2A* consistently inhibited *CDK4* over time across all three datasets. Our statistical analysis revealed that *CDK6* expression decreased over time, as did that of *MAP2K2*, a gene involved in neuronal autophagy, implying that the drug’s effect on GBM occurs via the GLIOMA pathway.

In conclusion, the antidepressant drug vortioxetine acts by inhibiting the proto-oncogene *JUN* while activating the oncogene *PIK3R5,* implying that the drug may not be effective enough to treat GBM in the ERBB signaling pathway. Another implication is that for vortioxetine to be effective in treating cancer, its potency in inhibiting *JUN* in the ERBB signaling pathway must be greater than its potency in activating *PIK3R5*. Nevertheless, further studies are required to elucidate the specific effects of vortioxetine [38]. The results of gene interactions in the GLIOMA pathway were more consistent than those in the ERBB pathway; the tumor suppressor *CALML6* was activated by eight genes, and the oncogene *CCND1* was inhibited by three genes. Although we do not have a clear explanation for this, our study findings indicate that the GLIOMA pathway, rather than the ERBB signaling pathway, may be responsible for the vortioxetine-induced anti-glioblastoma action. Further research is required to determine how the two signaling pathways interact in vortioxetine-induced GBM.

## Figures and Tables

**Table 1:** Overexpressed genes indicate increased expression over time, while underexpressed genes indicate decreased expression over time in the ERBB signaling pathway.

Overexpressed Genes	Difference	*p* Value	Underexpressed Genes	Difference	*p* Value
*BRAF*	1.30	0.325	*MAP2K2*	−1.92	0.010
*CRKL*	1.21	0.186	*AKT1*	−1.00	0.198
*CBLB*	1.15	0.213	*RPS6KB2*	−1.40	0.193
*HBEGF*	1.60	0.176	*TGFA*	−1.01	0.446
*CRK*	1.96	0.116	*SHC1*	−1.25	0.212
*ERBB3*	1.36	0.235	*PTK2*	−1.12	0.210
*GRB2*	1.00	0.068	*NRG1*	−1.01	0.239
*NRG2*	1.01	0.372	*CAMK2D*	−1.19	0.197
*AKT2*	1.22	0.187	*JUN*	−3.02	0.095
			*SRC*	−1.09	0.187

**Table 2:** Overexpressed genes indicate increased expression with time, while underexpressed genes indicate decreased expression with time for the GLIOMA pathway.

Overexpression Genes	Difference	*p* Value	Underexpressed Genes	Difference	*p* Value
*PDGFA*	1.02	0.258	*AKT1*	−1.00	0.198
*AKT2*	1.22	0.188	*TGFA*	−1.01	0.446
*BRAF*	1.30	0.325	*SHC1*	−1.26	0.212
*MDM2*	1.29	0.418	*EGFR*	−1.12	0.210
*GRB2*	1.00	0.068	*CDK6*	−1.01	0.444
			*CCND1*	−1.09	0.137
			*CAMK2D*	−1.19	0.197
			*MAP2K2*	−1.92	0.010

## Data Availability

The dataset GSE 214968 is available at https://www.ncbi.nlm.nih.gov/geo/query/acc.cgi?acc=GSE214968 accessed on 16 January 2025 and GSE214965 at https://www.ncbi.nlm.nih.gov/geo/query/acc.cgi?acc=GSE214965 accessed on 16 Jan. 2025. KEGG pathway information was downloaded from https://www.gsea-msigdb.org/gsea/downloads.jsp accessed on 1 February 2025. The original contributions presented in this study are included in the article. Further inquiries including code and genes beyond the top 10 can be directed to the corresponding author.
